# Paper vs. Pixel: Can We Use a Pen-and-Paper Method to Measure Athletes' Implicit Doping Attitude?

**DOI:** 10.3389/fpsyg.2017.00876

**Published:** 2017-06-12

**Authors:** Derwin K. C. Chan, Alfred S. Y. Lee, Tracy C. W. Tang, Daniel F. Gucciardi, Patrick S. H. Yung, Martin S. Hagger

**Affiliations:** ^1^School of Public Health, University of Hong KongPokfulam, Hong Kong; ^2^Health Psychology and Behavioural Medicine, School of Psychology and Speech Pathology, Curtin UniversityPerth, WA, Australia; ^3^School of Physiotherapy and Exercise Science, Curtin UniversityPerth, WA, Australia; ^4^Department of Orthopaedics and Traumatology, Chinese University of Hong KongShatin, Hong Kong; ^5^Faculty of Sport and Health Sciences, University of JyväskyläJyväskylä, Finland; ^6^Department of Physical Education, Hong Kong Baptist UniversityHong Kong, Hong Kong

**Keywords:** implicit association test, paper-and-pen IAT, automatic awareness to doping, banned performance-enhancing substances, prohibited drugs in sport

## Paper vs. pixel: can we use a pen-and-paper method to measure athletes' implicit doping attitude

Doping attitude is an individual's subjective evaluation (e.g., good or bad, useful or useless) toward the use of prohibited performance-enhancing substances or methods in sports. Research on doping attitude has traditionally relied on self-report questionnaire methods to measure the construct (Ntoumanis et al., 2014; Chan et al., 2015). However, as doping in sport is illegal (World Anti-Doping Agency, 2015) and perceived as socially unacceptable, athletes who hold positive attitudes toward doping are less likely to reveal them to others. As a result explicit measures of doping attitude are susceptible to potential bias as athletes may respond in a socially desirable fashion (Petróczi and Aidman, 2009; Gucciardi et al., 2010). To counter such bias, implicit measures such as the implicit association test (IAT; Greenwald et al., 1998) have been developed to capture individuals' non-conscious attitudes toward doping (Brand et al., 2014a,b; Schindler et al., 2015). The current paper aims to introduce a paper-and-pen IAT which could potentially serve as alternative method to the traditional computer-IAT for measuring athletes' doping attitude.

## Traditional computer-IAT and paper-and-pen IAT

The IAT is a timed decision task which measures individuals' response latencies in sorting competing sets of stimuli. Stimuli can be in the form of words (Petróczi et al., 2008) or pictures (Brand et al., 2014a). In a traditional IAT, there are seven blocks. In each block participants are given a single stimulus, and are asked to categorize the stimulus according to word-pairs representing combinations of dichotomous superordinate *concept* (e.g., fruit vs. snack) and *attribute* valence categories (e.g., like vs. dislike; Petróczi et al., 2011; Brand et al., 2014b; Schindler et al., 2015). However, as some topics do not have obvious dichotomous complementary categories (i.e., doping), researchers have also developed a four-block single-category IAT (SC-IAT) consisting of a *single* concept category, and two attribute categories (Karpinski and Steinman, 2006; Chan et al., 2017). The SC-IAT design is advantageous in measuring implicit doping attitude as the concept of “doping” does not have a clear contrast category. For example, previous research using IAT to measure doping attitudes has used “nutritional supplements” as the contrast category to “doping,” but as nutritional supplements sometime contain banned substances, it is a problematic contrasting category for the IAT (Brand et al., 2011). To take it even further, Backhouse et al. (2011) suggested a “gateway hypothesis” which explains why athletes, who choose to use legal performance enhancing substances (through nutritional supplement consumption not banned by the World Anti-Doping Agency), can also encompass a subgroup of “at-risk” athletes for future illegal doping practices. Moreover, those athletes who consume legal doping substances may/may not be entirely able to distinguish between legal and illegal supplements within a short reaction timed IAT. Henceforth, an SC-IAT may serve as a solution through the examination of doping as a single concept category alongside the two *attribute* valence categories. In the critical blocks of the SC-IAT, the target concept (e.g., doping) is matched with positive (like) or negative (dislike) attributes. The response time needed for participants to correctly categorize the stimuli into like/dislike attributes provides an estimate of the individual's automatic association strength between the stimuli and the concept-attribute combination. Implicit doping attitude can then be inferred by subtracting scores between contrasting concepts/attributes. The attitude will be overall positive if responses are faster to trials in which doping is paired with positive attribute stimuli compared to trials when doping is paired with negative attribute stimuli.

Traditionally, the IAT is conducted on computer (Petróczi et al., 2008; Brand et al., 2014a; Schindler et al., 2015) using specialist experimental software (e.g., E-Prime). Although computer-based data collection is preferred, there are situations where it is impractical or infeasible (i.e., sporting field or swimming pool to test athletes). Furthermore, the availability of a computer and software to administer computer-IATs can restrict the number of participants tested simultaneously, making data-collection time-consuming and placing constraints on study design and geographical location of sampling. Although recent developments of online versions of computer-based IAT may offer more flexibility in data-collection, they could be costly and heavily dependent or influenced by distractions in the environment. For data collection in these situations, researchers have developed a protocol for a paper-and-pen IAT as an alternative to computer-based measures (Lemm et al., 2008). Running paper-and-pen IATs places less demands on the availability of specialized equipment and gives the researcher options to collect data in the field and administer it simultaneously to a large groups of participants. The pen-and-pen IAT, therefore, affords greater flexibility studies that require larger sample sizes, on-site administration, or access to specific samples that cannot attend a laboratory.

In a paper-and-pen IAT, responses are made by marking a circle either on the left or the right of the stimulus instead of pressing left or right keys on the keyboard. The strength of automatic association is quantified by the number of correct responses within a given period of time (e.g., 20 s in each trial). Although paper-and-pen IAT does not measure the precise reaction time for each stimulus within a block, it provides a close approximation of a respondent's global reaction time in each block; hence, given situational factors, it can be a plausible alternative to the computer-IAT.

Importantly, the paper-and-pen IAT shares the same underlying protocol as a computer-based IAT, with response patterns on the paper-and-pen versions showing to be consistent with the computer versions in multiple contexts (Lowery et al., 2001; Teachman and Brownell, 2001; Teachman et al., 2003; Sinclair et al., 2005) including disability (Pruett and Chan, 2006; Dionne et al., 2013), death (Bassett et al., 2003) and smoking (Bardin et al., 2016). Researchers have emphasized the convenience and cost effectiveness of the paper-and-pen IAT, as it is inexpensive, time-saving, and can be administered simultaneously to large groups of participants (Teachman et al., 2003; Vargas et al., 2007; Lemm et al., 2008). For these reasons, we believe there is potential for the paper-and-pen IAT to be an alternative method for measuring athletes' implicit doping attitude when computerized IAT is infeasible. Yet, the paper-and-pen IAT is a relatively under-used methodological approach and has yet to be used in a doping context. To stimulate such research, we propose a paper-and-pen IAT protocol designed to measure athletes' implicit attitude toward doping.

## Paper-and-pen IAT protocol in doping

**Figure 1:**
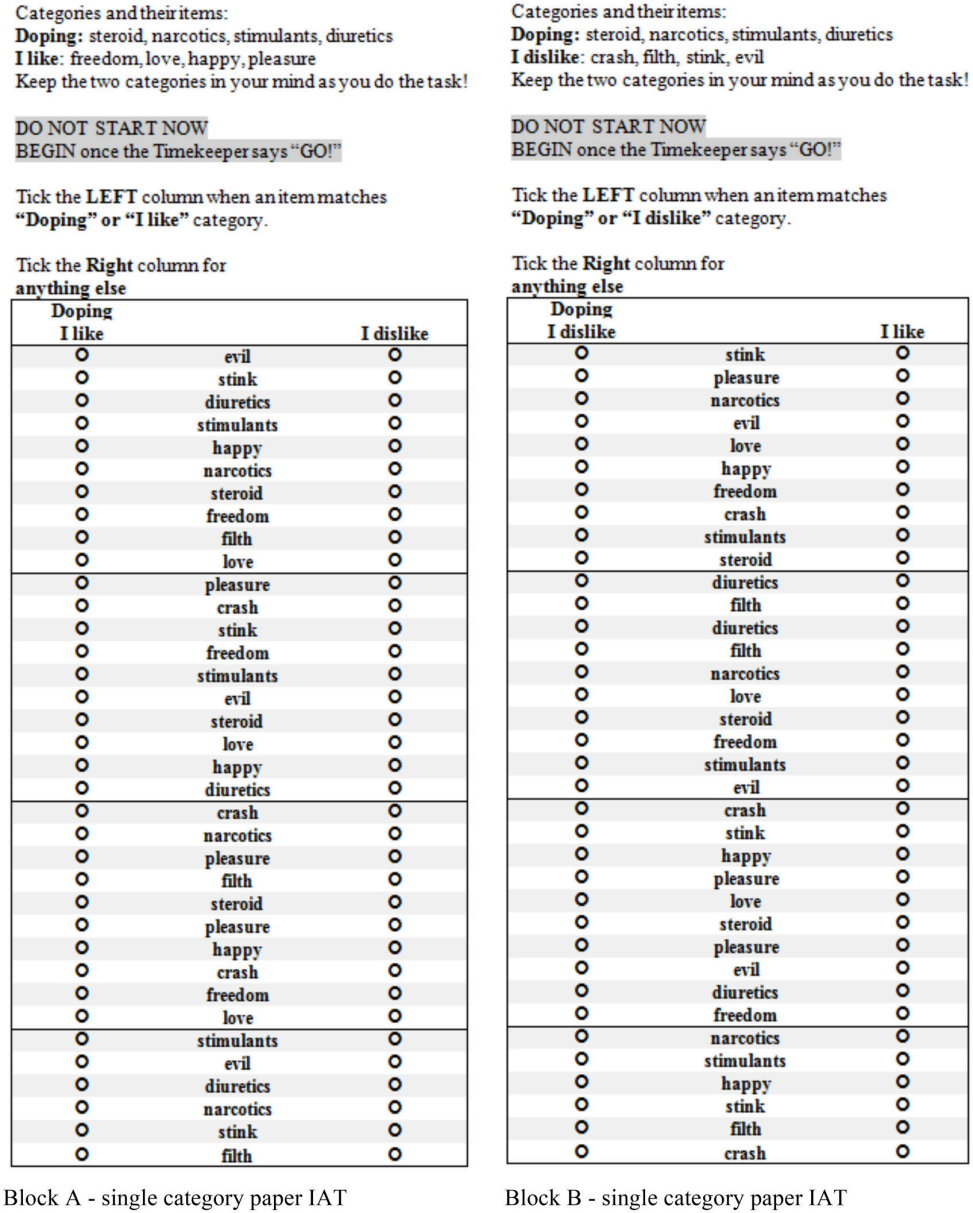
Block (**A**,left) and Block (**B**, right) of paper-and-pen IAT for implicit doping attitude.

The proposed paper-and-pen IAT protocol is based on the existing protocol of the single-category IAT (Karpinski and Steinman, 2006) and has been adapted to measure implicit doping attitudes (Chan et al., 2017). The IAT is a modified form of the traditional computer-format single-category IAT that requires participants to categorize stimuli into a single concept category: doping (i.e., steroid, narcotics), or the two attribute categories of “I like” (i.e., freedom, love), or “I dislike” (i.e., crash, filth). Similarly, the paper-and-pen IAT comprises two blocks each consisting of two focal categories (e.g., “doping” or “I like”) and a single non-focal category (e.g., “I dislike”). The respondents' task is to indicate as quickly and accurately as possible within 20 s which category the stimuli falls into by marking the radio buttons left or right (see Figure 1). In Block A, the focal category is “doping” or “I like”, however, this target switches to “doping” or “I dislike” in Block B in order to accommodate associations between the concept category and both attribute categories. It is recommended that presentations of the blocks are counterbalanced and that participants be offered a practice trial before each test block to familiarize them with the procedure and categories. The number of correct responses to the items in Blocks A and B provide an indication of the strength of automatic associations between the concept (“doping”) and attributes (“I like”/“I dislike”) and are used to calculate a response latency representing the implicit attitude, the D-score.

According to Lemm et al. (2008), there are several scoring algorithms to compute the D-score: simple difference score (A–B), simple ratio (A/B–1), latency conversion (1,000^*^((1/B)–(1/A))), and product: square root of difference (if A > B, A/B^*^√|A–B|; if B > A, ratio = B/A^*^(−1)^*^√|A–B|). A validation study showed that the product: square root of difference is the algorithm that produced the D-score to closest to that generated by the computer-based IAT (Lemm et al., 2008); hence, it has been the most frequently used computation method (Teachman et al., 2003; Lane et al., 2005; Pruett and Chan, 2006; Dionne et al., 2013).

## Conclusion

Implicit association test is a useful tool for measuring athletes' implicit attitude toward doping. Traditionally, implicit attitude test heavily relies upon computers, but recent development of IAT has brought-forth a paper-and-pen IAT that can serve as an alternative tool in assessing athletes' implicit doping attitude. In comparison to computer-IATs, paper-and-pen IATs are less-costly, more convenient and efficient to run, and offer greater flexibility in terms of study design and location of data-collection. Although paper-and-pen IATs have received growing amount of support in the fields of social and health psychology (Bardin et al., 2016), additional research is required to examine validity and reliability of our proposed paper-and-pen IAT against a computer-based IAT in the context of doping.

## Author contributions

All the authors contribute intellectually to the conceptualization and the actually writing-up of this opinion paper.

### Conflict of interest statement

The authors declare that the research was conducted in the absence of any commercial or financial relationships that could be construed as a potential conflict of interest. The reviewer DL and handling Editor declared their shared affiliation, and the handling Editor states that the process nevertheless met the standards of a fair and objective review.
